# Microstructural Evolution, Mechanical Properties, and Preosteoblast Cell Response of a Post-Processing-Treated TNT5Zr β Ti Alloy Manufactured via Selective Laser Melting

**DOI:** 10.1021/acsbiomaterials.1c01277

**Published:** 2022-05-10

**Authors:** Weihuan Kong, Sophie C. Cox, Yu Lu, Victor Villapun, Xiaoling Xiao, Wenyou Ma, Min Liu, Moataz M. Attallah

**Affiliations:** † School of Materials and Metallurgy, University of Birmingham, Edgbaston, Birmingham B15 2TT, U.K.; ‡ School of Chemical Engineering, University of Birmingham, Edgbaston, Birmingham B15 2TT, U.K.; § Guangdong Institute of Analysis, Guangzhou 510651, PR China; ∥ Guangdong Institute of New Materials, Guangzhou 510651, PR China

**Keywords:** selective laser melting, β Ti alloy, post-processing treatment, strength-to-modulus ratio, fatigue properties, biocompatibility

## Abstract

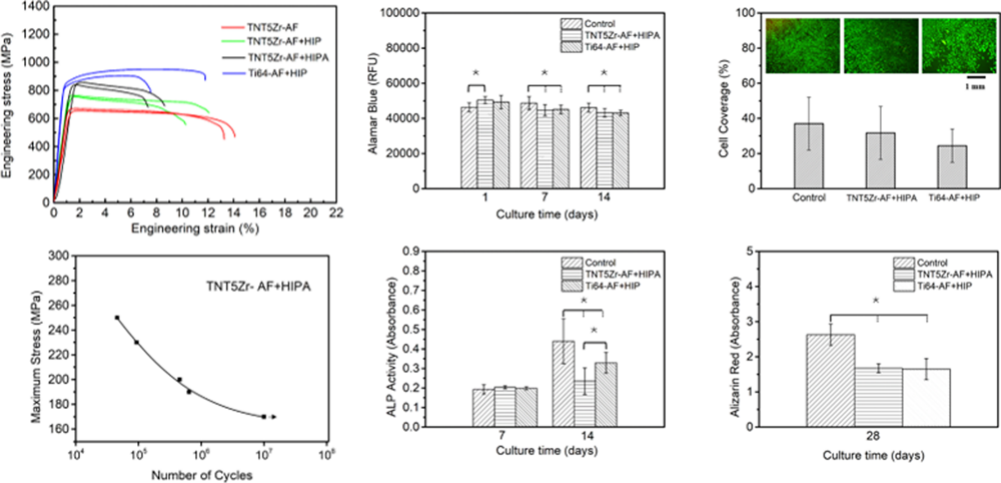

A Ti–34Nb–13Ta–5Zr (TNT5Zr) β Ti alloy with a high strength-to-modulus ratio has been developed, showing its potential to become another candidate material in load-bearing implant applications. This work mainly investigates the microstructural evolution, mechanical properties, and biocompatibility of a post-processing-treated TNT5Zr alloy manufactured via selective laser melting (SLM). Transmission electron microscopy observation shows the existence of the single beta grain matrix and alpha precipitates along the grain boundary in the SLM + HIP manufactured TNT5Zr alloy (TNT5Zr-AF + HIP), and ellipsoidal nano-sized intragranular α″ precipitates (approx. 5–10 nm) were introduced after the subsequent low-temperature aging treatment. The precipitation strengthening enables the SLM + HIP + aging manufactured TNT5Zr (TNT5Zr-AF + HIPA) alloy to show a comparable ultimate tensile strength (853 ± 9 MPa) to that of the reference material (Ti64-AF + HIP, 926 ± 23 MPa). Including the inferior notch-like surface of the test pieces, the slip-band cracking that occurs in this ductile TNT5Zr-AF + HIPA alloy is regarded as the main factor in determining its fatigue strength (170 MPa). *In vitro* short-term biocompatibility evaluation reveals almost no significant difference in the preosteoblast viability, differentiation, and mineralization between TNT5Zr-AF + HIPA and the reference biomaterial (Ti64-AF + HIP).

## 1 Introduction

Some snapshots in the scope of bone mechanics provide significant guidance in bone replacement research and development. Bone is a lightweight composite with a combination of collagen, minerals, and non-collagenous proteins.^1^ As an extraordinary multifunctional hard tissue, bone is responsible for mechanical support, aiding in mineral homeostasis, and hematopoiesis.^2^ Young’s modulus is one of the crucial mechanical properties of the human bone. It shows that the elastic modulus of the diaphyseal cortical bone people aged 53 to 93 years measured using the nanoindentation test was 20.1 ± 5.4 GPa and that of the cancellous bone was 11.4 ± 5.4 GPa.^3^ Osteoblasts, which are responsible for bone formation, are recognized by the site of the bone surface exhibiting high levels of alkaline phosphatase (ALP) and osteocalcin.

There are a high percentage of aging people suffering severe osteoarthritis worldwide. Therefore, it is necessary to develop satisfactory implant materials to replace malfunctioned load-bearing human joints. Historically, a variety of materials such as cobalt-based alloys, stainless steel, and titanium alloys have been developed for joint replacement. For example, Co–Cr–Mo alloys containing sufficient C (>0.20%) have been designed as an implant material because of the existence of wear-resistant carbides (e.g., M23C6-type).^4^ SS 316L stainless steel due to its low cost, availability, and good weldability has been regarded as an attractive candidate for joint replacement.^5^ When comparing Young’s moduli of these alloys to that of the natural bone, there exists a massive mismatch between them. It has been demonstrated that high-stiffness implants caused bone resorption due to the “stress shielding” effect.^6^ Meanwhile, some tissue reaction cases have been documented after the implantation of cobalt-based or nickel-based alloys. Released Cr and Co ions in Co–Cr–Mo alloys have been considered to increase the *in vivo* carcinogenic potential.^7,8^ It has also been mentioned that the nickel ion released from a stainless-steel implant caused a loss of cell viability.^9^

In the field of alloys with low Young’s modulus designed for load-bearing implants, some studies have been conducted on Ti–Nb-based β Ti alloys manufactured using traditional manufacturing techniques. Hanada et al.^10^ showed the feasibility of obtaining a low elastic modulus (approx. 40 GPa) after optimizing the composition of Ti–Nb–Sn β Ti alloys. They regarded that the Sn addition retards ω and precipitates transformation, thereby reducing its Young’s modulus. Laheurte et al.^11^ designed β Ti alloys with biofriendly elements (e.g., Nb, Ta, and Zr) using the DV-Xα electronic approach. They concluded that the incipient modulus of β Ti alloys can be reduced to a value as low as 30 GPa, which is extremely close to its counterpart of the cortical bone. Panigrahi et al.^12^ demonstrated the mechanical properties, microstructural, and textural evolution of the Ti–45Nb alloy manufactured via severe plastic deformation. They found that the elastic moduli of samples after hydrostatic extrusion and high-pressure torsion remained the same as that of the initial ingot at the level of 65 GPa, hypothesizing that the volume fraction of the ω phase induced by deformation is too small to influence its elastic modulus.

Besides the material’s elastic modulus, biocompatibility is another important factor in load-bearing implant development. Gordin et al.^13^ and Neacsu et al.^14^ demonstrated that Ti–Nb–X alloys possess equal or even better short-term *in vitro* MC3T3-E1 preosteoblast response when compared with that of commercial pure titanium. Miura et al.^15^ and Ion et al.^16^ evaluated the preosteoblast cell response of Ti–25Nb–11Sn, Ti–23Nb–0.7Ta–2Zr–0.5N (wt %), and Ti–6Al–4V alloy. The two research groups both observed the same level of preosteoblast attachment, spreading, and proliferation in these biomaterials.

Powder bed fusion has become capable of manufacturing tailored parts with dimensional accuracy after several decades of technological development.^17,18^ It also shows relatively lower feedstock consumption than other manufacturing techniques. It may make the implant more affordable if the manufactured biofriendly β Ti alloys consisted of precious elements (e.g., Nb, Ta, and Zr). Combining the aforementioned benefits of Ti–Nb-based β Ti alloys (e.g., narrow elastic modulus mismatch to the natural bone and excellent biocompatibility), it is necessary to consider what the main disadvantages of Ti–Nb-based β Ti alloys obtained via additive manufacturing (AM) are. The first concern arises from the relatively low ultimate tensile strength (UTS) caused by the main beta phase matrix with the body-centered cubic (bcc) lattice arrangement, which is observed in high β-stabilized Ti alloys when undergoing a relatively high cooling rate. Recently, Ummethala et al.^19^ and Kong et al.^20^ manufactured single β phase Ti–35Nb–7Zr–5Ta (wt %) and main β phase Ti–34Nb–13Ta–5Zr (wt %) using selective laser melting (SLM), and the UTSs collected from the tensile test were 631 MPa and 694–702 MPa, respectively. Therefore, it increases the risk of early-stage failure for the implant in service. Another concern is that the existence of the as-fabricated keyhole due to localized element evaporation may deteriorate the material’s mechanical properties.^21,22^ Hot isostatic pressing (HIP) is a technique that involves simultaneous functions of elevated temperature and high pressure applied with an inert argon gas in a specific vessel. Argon gas is pressed on every surface of a component in a normal direction like “hot forge”, and then, densification is fulfilled due to a surface energy reduction of the pores.^23^ In this study, we investigated the microstructural evolution, defects distribution, and mechanical properties of the Ti–34Nb–13Ta–5Zr β Ti alloy before and after the post-processing treatment, such as traditional HIP and the low-temperature aging treatment. In addition, we investigated the short-term *in vitro* MC3T3-E1 preosteoblast response of the post-processed β Ti alloy. The Ti–6Al–4V alloy, which is commonly found in load-bearing biomedical implants, was inserted as a benchmark to compare the tensile properties and cell culture results.

## 2 Materials and Methods

### 2.1 Powder Feedstock

**Figure 1:**
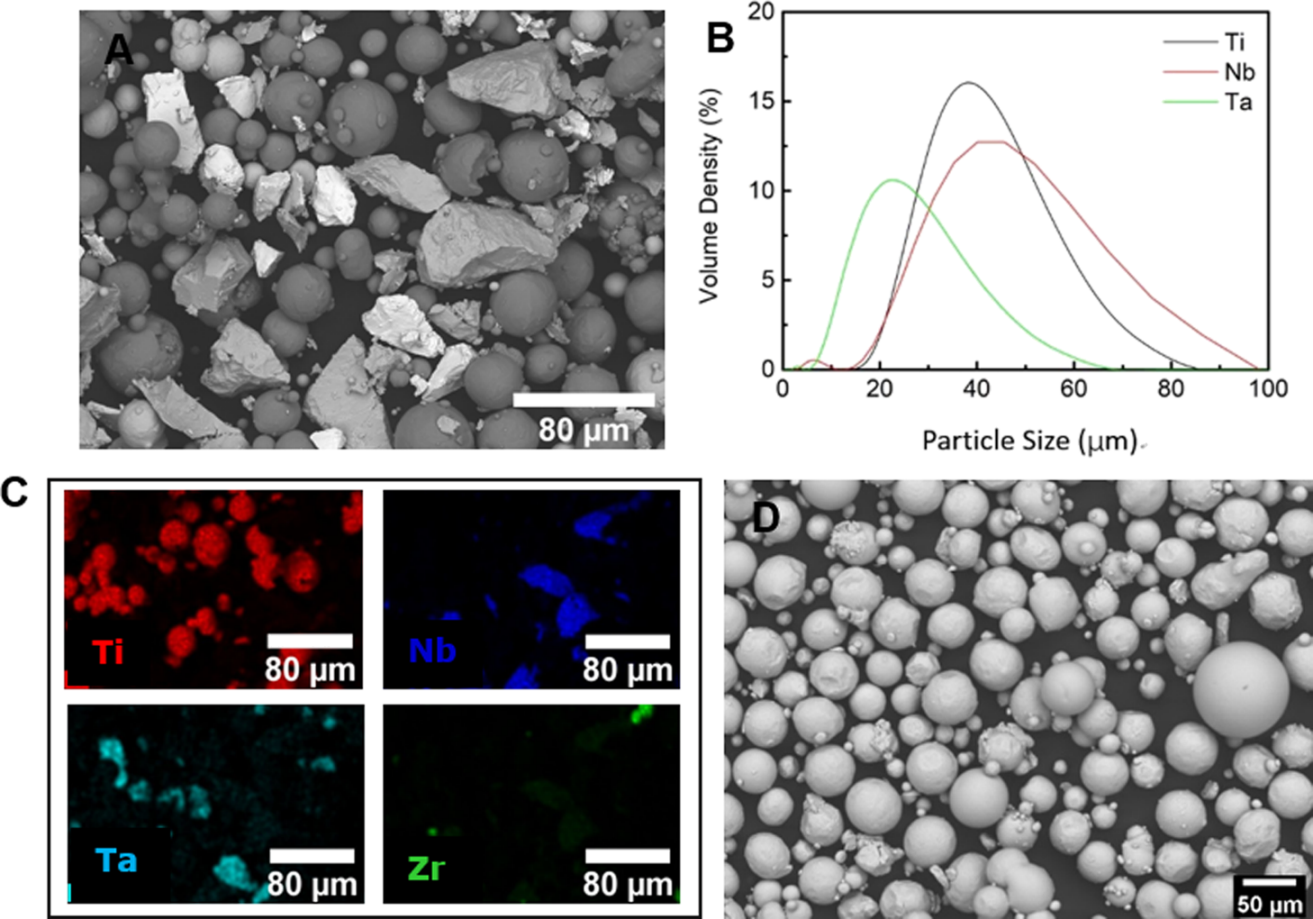
(A) Morphology of the TNT5Zr powder after blending, (B) particle size distribution of the as-received pure element powders, and (C) EDS map after blending. (D) Morphology of Ti–6Al–4V pre-alloyed powder. Note: the particle size distribution of Zr powder is not listed as it is too reactive to be measured.

Pure element (Ti, Nb, Ta, and Zr) powders were used for mixing the as-designed material powder feedstock. The chemical composition and particle size of the Ti–34Nb–13Ta–5Zr alloy are given in Table 1, which is hereafter termed as the TNT5Zr alloy. The spherical Ti and Zr powders (TLS, Germany) with particle size ranges of 15–83 μm and 10–45 μm, respectively, were gas-atomized in an argon atmosphere. The rocky Nb (Elite, UK) and Ta powders (H.C. Starck, Germany) with particle size ranges of 5–86 μm and 8–52 μm (Figure 1B), respectively, were manufactured using the hydride–dehydride process. The mass of each elemental powder was measured using a top pan balance (Kern EMB2000, 0.01 g accuracy) inside a glovebox (Saffron, UK) with an argon protective atmosphere (≤0.005% O2). Then, the powder was mixed for 10 h in a horizontal rotating drum (Kimber-Allen, UK). The blended powder was characterized using an energy-dispersive X-ray spectroscopy (EDS, Bruker) map for checking the mixing performance before the manufacturing process (Figure 1A,C). The pre-alloyed Ti–6Al–4V powder with a particle size range of 20–65 μm manufactured using gas atomization was used for building the reference specimens (Figure 1D).

**Table 1:** Chemical Composition of the TNT5Zr Alloy and Particle Size of the Involved Pure Element Powders for SLM

	Ti	Nb	Ta	Zr
composition (wt %)	Bal	34	13	5
particle size (μm)	15–83	5–86	8–52	10–45

### 2.2 Selective Laser Melting

An M2 Cusing SLM system (Concept Laser, GE Additive) was adopted to fabricate TNT5Zr samples on a Ti alloy substrate. The machine was equipped with a 400 W Yb:YAG fiber laser at a wavelength of 1064 nm. The laser beam spot size was focused to be approx. 63 μm. Because of the high oxidation risk caused by these pure element powders, high-purity protective argon was continually supplied inside the SLM build module until the components were thoroughly cooled down. The optimized SLM parameter for manufacturing specimens were as follows: 300 W laser power, 500 mm/s scanning speed, and 50 μm scanning spacing. The scan strategy was chessboard with scan vectors rotated by 90° in adjacent 5 × 5 mm blocks, where each successive layer was designed by shifting 1 mm in both *X* and *Y* directions; the preset layer thickness was 20 μm. The manufactured parts were 7 × 7 × 7 mm cubes and sub-size dog bone tensile and fatigue specimens with a 75 mm total length. The tensile specimen dimensions were designed according to the ASTM-E8/E8M-13a standard;^24^ the fatigue specimen was designed with tangentially blending fillets between the uniform test section and the ends according to the ASTM-E466-15 standard.^25^ The tensile and fatigue parts (10 mm in height) were horizontally manufactured on the SLMed original side surface and then sliced into dog bone test pieces (1.5 mm in height) using a wire electron discharge machining (GF Machining Solutions) system. A manual grinding kit (8220, Dremel) was used to reduce the surface roughness of fatigue test pieces.

### 2.3 Post-Processing Heat Treatment and Porosity Distribution

Traditional HIP (EPSI) was used to close pores induced by SLM. The process parameters were as follows: 3 h of dwelling time at 1000 °C in the container filled with 120 MPa pressurized argon, followed by furnace cooling with a 10 °C/min cooling rate. A low-temperature (300 °C) aging treatment with different aging times (1, 4, 7, 10, 13, 24, 48, 64, and 72 h) was investigated to strengthen the TNT5Zr alloy. The finalized aging time (48 h) for microstructural evaluation, mechanical tests, and *in vitro* tests was decided according to the microhardness aging time results. The porosity and unmelted particle of samples (1.1 × 1.1 × 7.5 mm) before and after the HIP treatment were analyzed using microcomputed tomography (micro-CT) (Skyscan, Bruker). The scan parameters were as follows: an accelerating voltage of 165 kV and a current of 75 μA for a 360° scan. A total of 2500 projections were collected on a charge-coupled device detector using a 1s exposure time. The data were reconstructed and visualized using Nikon Pro 3D and Avizo software, respectively.

### 2.4 Microstructure Characterization

Metallographic specimens were prepared using an automatic grinding and polishing machine (Tegramin 30, Struers) and then etched using Kroll’s solution (2% HF + 6% HNO_3_ + 92% H_2_O). A field emission scanning electron microscopy (SEM) gun (JSF-7000F, JEOL) was employed for observing the microstructure. The phase identification was performed using an X-ray diffractometer (AXRD, Proto) with Cu Kα radiation, and the X-ray diffraction (XRD) spectra were collected with fixed parameters of a 0.02° step size and a 2s time/step. To reveal the evolution of the texture, the samples before and after HIP were examined via electron backscatter diffraction (EBSD) using a scanning electron microscope (NNS450, FEI). The step size was 0.4 μm, aiming to obtain a high EBSD indexing rate for the characterization of the texture and grain size. A transmission electron microscope (2100, JEOL) operating at 200 kV was used to capture the bright-field (BF) images and selected area diffraction (SAD) patterns, BF scanning transmission electron microscopy (BF-STEM) images, and high-resolution TEM (HRTEM) images. Thin foils for TEM were prepared through an argon ion milling technique (Gatan PIPS, Ametek), involving gradient milling using different Ar ion energy and sputter angle settings.

### 2.5 Mechanical Test

Mirror-like specimens were mounted on a micro-hardness tester (Wilson VH1202, Buehler) for Vickers hardness measurement. The test for each sample was performed with a 100 g load and 10 times linearly indented with the recommended spacing according to ASTM E384-17.^26^ Tensile testing was carried out with specimens (1.6 × 6 mm rectangular cross-section and SLM-processed original side surface) placed perpendicularly to the build direction at room temperature. The stress–strain curves were measured at a crosshead speed of 0.5 mm/min at room temperature using a tensile testing machine (2500, Zwick/Roell). Two specimens per alloy were tested in order to obtain the average tensile properties, and a clip-on extensometer was attached to a specimen of 15 mm gage length until rupture. The fatigue test was carried out on specimens with a rectangular cross-section of 1.5 × 6 mm placed perpendicularly to the build direction. Axial high cycle fatigue testing was performed on a fatigue testing machine (Vibraphore Resonant) using magnetic resonance to deliver a low constant amplitude, a load ratio of *R* = 0.1, and a high frequency (usually in a range of 50–100 Hz, decided from the specimen geometry and stiffness). The testing with a maximum stress (up to 250 MPa) inside the elastic region was performed at room temperature. The tensile and fatigue fracture morphology was observed using a scanning electron microscope (JSF-7000F, JEOL).

### 2.6 *In Vitro* MC3T3-E1 Performance

MC3T3-E1 preosteoblasts were selected to evaluate the cell response of TNT5Zr and Ti–6Al–4V alloys. All metallic specimens were ground using 1200 Grit SiC sandpaper for 10 min. Then, they were ultrasonically cleaned in pure ethanol for 10 min and then autoclaved at 121 °C for 90 min before cell seeding. All metallic substrates (10 × 5 mm) and polyester plastic coverslip controls (ThermoFisher, D13 mm) were placed in a 24-well plate and seeded with preosteoblast cells at a density of 2 × 10^4^ cells/cm^2^. Samples were incubated at 37 °C in a humidified atmosphere with 5% CO_2_. The cell culture medium was the minimal essential medium (MEM) supplemented with 10% fetal bovine serum, 1% penicillin/streptomycin, and 0.5 g/L l-glutamine. The medium was changed every 2 days during the specific culture periods. Cell viability was evaluated via Alamar blue staining after 1 day, 7 days, and 14 days of culture using a spectrophotometer (Spark, Tecan) at a 560 nm excitation wavelength and a 590 nm emission wavelength. A calcein-AM and propidium iodide solution was chosen to evaluate the viability of MC3T3-E1 cells after 7 days of culture on the metallic and control substrates. Stained cells were visualized using a microscopic imaging system (EVOS M5000, Thermo Scientific). Nine optical microscopy images were captured using a 4× low magnification objective, and live cell coverage on each substrate was analyzed using image processing software (Image J, Fiji). The ALP level was measured using a SensoLyte pNPP ALP assay ait (AnaSpec Inc., US) for the 7 and 14 day cultured substrates, and the absorbance was read at a wavelength of 405 nm (Spark, Tecan). Total calcium deposits after 28 days of culture was studied using an Alizarin red staining (ARS) assay. Samples were fixed with 10% paraformaldehyde solution for 30 min and washed with deionized water three times. Then, 1 mL of cetylpyridinium chloride was added, and samples were cultivated for 1 h at 37 °C. The resulting elution was collected in a 96-well plate, and the absorbance was read at a wavelength of 570 nm (Spark, Tecan). The aforementioned assays were conducted in triplicate. All involved data were recorded as the mean ± standard deviation. Analysis of variance and two-tailed t-tests were performed for the as-mentioned assays, with a *p* value <0.05 considered as being statistically significant.

## 3 Results

### 3.1 Porosity and Unmelted Particle Distribution

**Figure 2:**
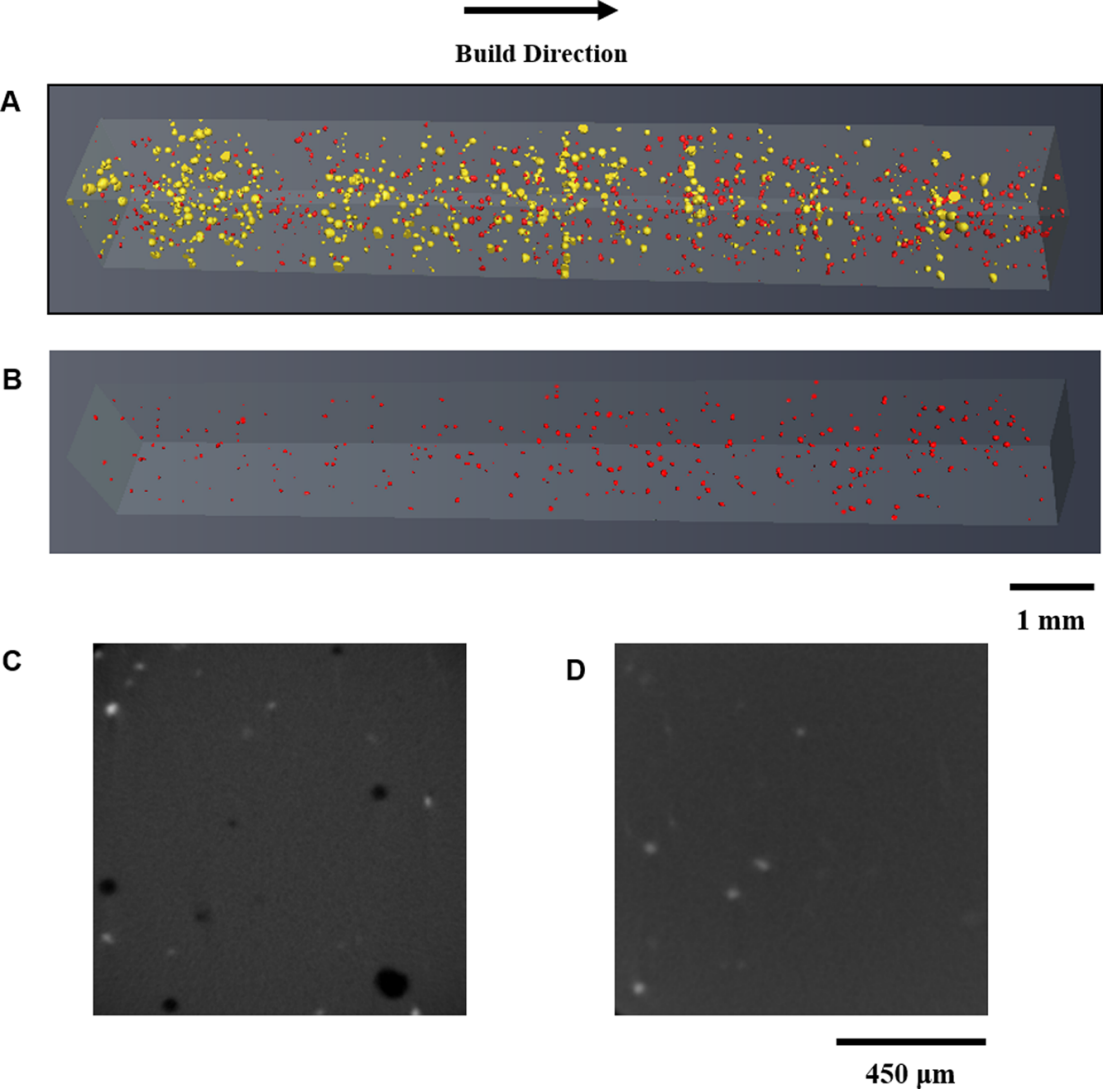
3D tomography of the unmelted Ta and Nb particles (red) and porosity (yellow) of the TNT5Zr samples: (A) as-fabricated and (B) after HIP at 1000 °C for 3 h. 2D reconstructed cross-sections of (C) as-fabricated and (D) as-HIPed TNT5Zr samples. The dark regions indicate pores, while the brighter regions indicate unmelted particles.

Figure 2A,B shows the reconstructed SLM-manufactured 3D parts before and after HIP obtained using the aid of the micro-CT technique. Micropores (yellow) with an irregular shape were randomly distributed in the as-fabricated TNT5Zr specimen (Figure 2A), hereafter termed TNT5Zr-AF. Figure 2B shows that no micropores can be detected in the sample after HIP treatment (hereafter termed TNT5Zr-AF + HIP). The low volume percentage of unmelted particles (red) can be found distributed in the scanned volume of both TNT5Zr-AF and TNT5Zr-AF + HIP specimens. Figure 2C,D shows the 2D cross-sectional micrographs of TNT5Zr in both conditions. Compared with the TNT5Zr-AF sample, the pores disappeared, and a low percentage of unmelted particles (Ta and Nb) remained in the sample after HIP.

### 3.2 Microstructure Characteristics

**Figure 3:**
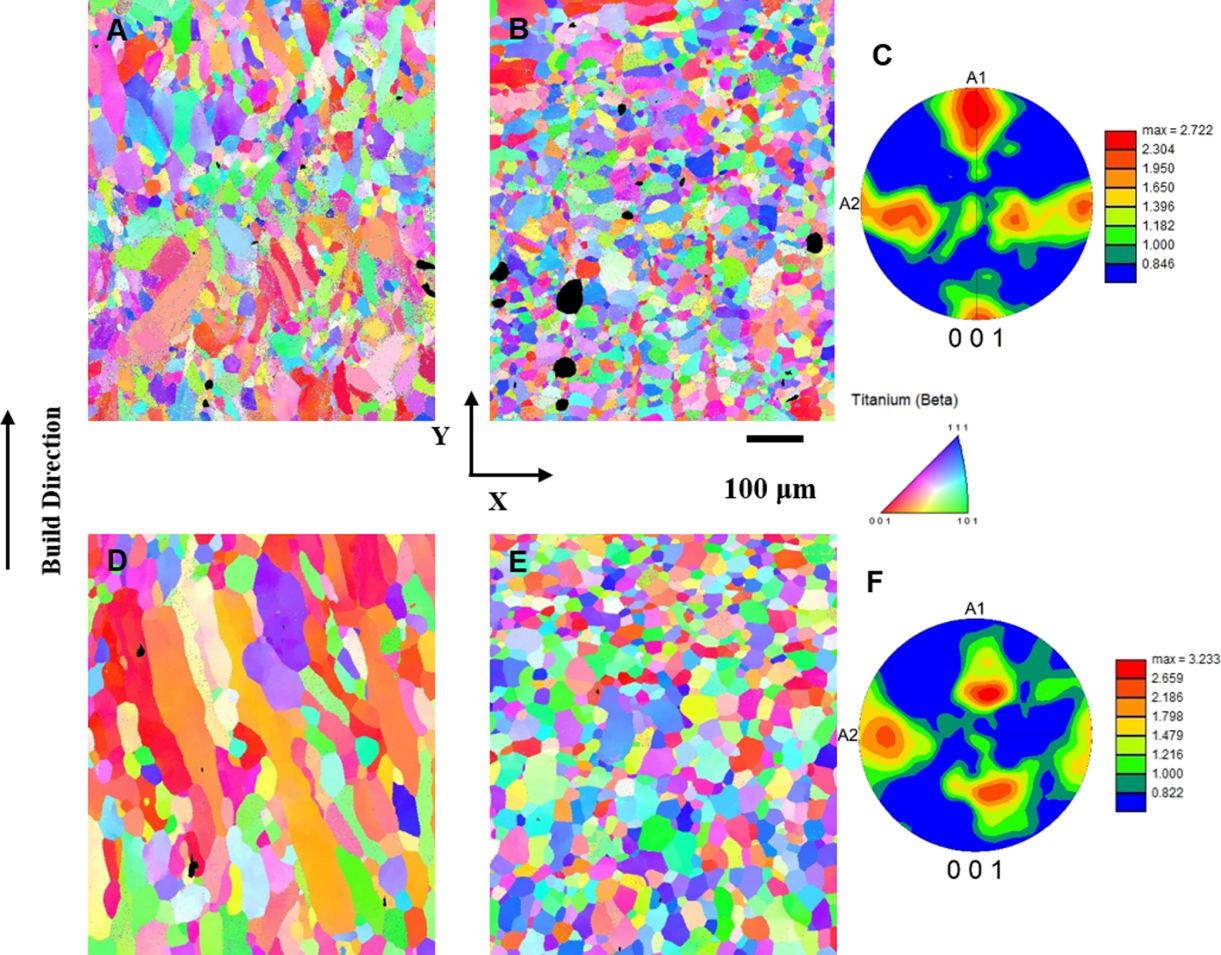
IPF map of TNT5Zr-AF (A) along the build direction, (B) along the perpendicular build direction, and (C) its pole figure (PF). IPF map of TNT5Zr-AF + HIP (D) along the build direction, (E) along the perpendicular build direction, and (F) its pole figure (PF).

The EBSD inverse pole figure (IPF) results of TNT5Zr-AF from the parallel and perpendicular build directions are shown in Figure 3A,B. It can be found that the columnar and equiaxed beta grains were obtained during SLM. Additionally, keyholes were observed in the TNT5Zr alloy manufactured via *in situ* alloying. The ⟨001⟩ fiber texture (Figure 3C) was formed because the grains grew preferentially along the ⟨001⟩ orientation, which is consistent with the main heat dissipation direction of the layer-by-layer SLM technique. The IPFs of TNT5Zr-AF + HIP from the parallel and perpendicular build directions (Figure 3D,E) show the same grain type and a phenomenon of grain growth when compared with those of the as-fabricated samples. The extent of the ⟨001⟩ preferred crystallographic orientation in the HIP-manufactured sample is much weaker than the counterpart of TNT5Zr-AF, as seen in Figure 3F.

**Figure 4:**
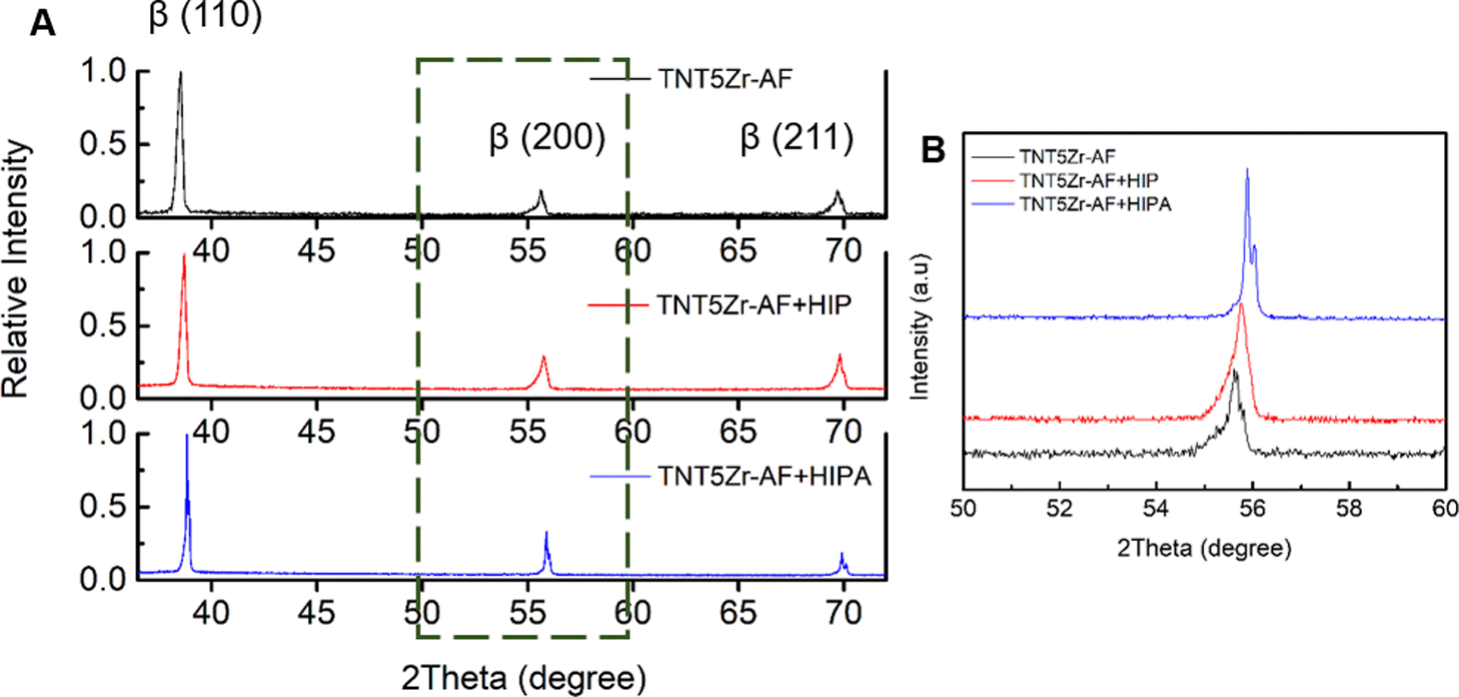
(A) XRD spectra of SLM manufactured TNT5Zr (TNT5Zr-AF), SLM + HIP manufactured TNT5Zr (TNT5Zr-AF + HIP), and SLM + HIP + aging manufactured TNT5Zr (TNT5Zr-AF + HIPA). (B) XRD profiles of as-mentioned specimens, where 2θ is located between 50 and 60°.

**Figure 5:**
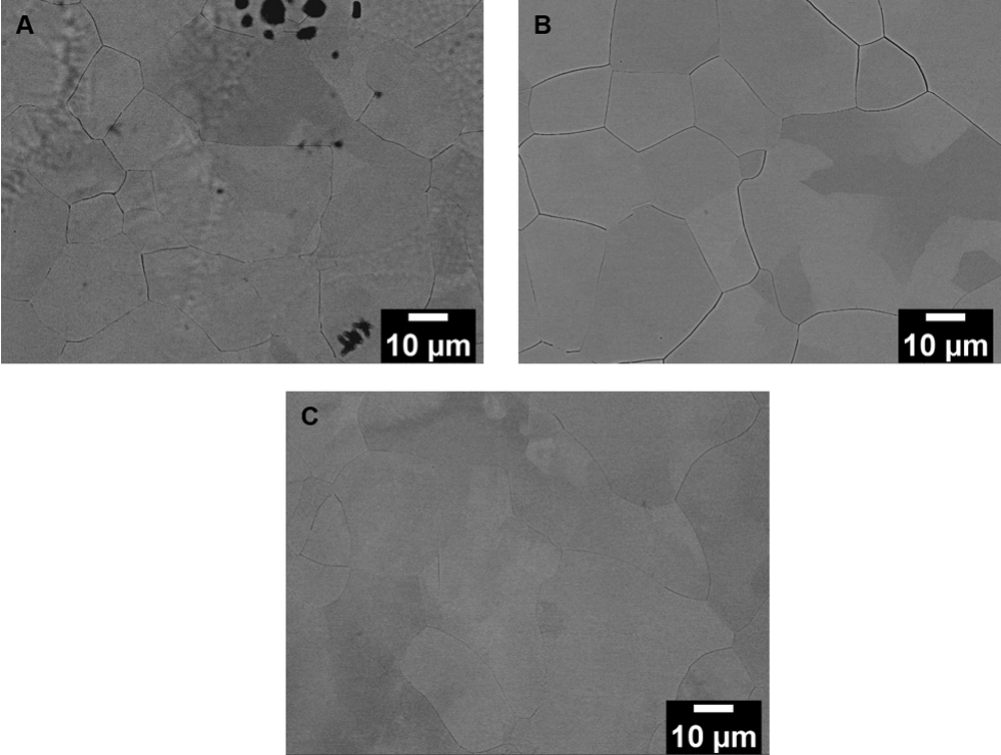
SEM micrographs of (A) TNT5Zr-AF, (B) TNT5Zr-AF + HIP, and (C) TNT5Zr-AF + HIPA.

The XRD profiles of different types of manufactured TNT5Zr are shown in Figure 4A. XRD patterns at a diffraction angle range of 36–72° demonstrate the main diffraction peaks of the beta phase. Figure 4B reveals that the SLM + HIP + aging manufactured TNT5Zr alloy (hereafter termed TNT5Zr-AF + HIPA) exhibits peak splitting along beta phase (200) due to the precipitation during low-temperature aging. The diffraction angle shift observed in post-processed TNT5Zr alloys may be relevant to the grain growth in thermal treatments. The corresponding SEM micrographs of TNT5Zr alloys are shown in Figure 5. The equiaxed beta grains were obtained in high β-stabilized TNT5Zr alloys without the presence of acicular α′ precipitates in the beta matrix.

**Figure 6:**
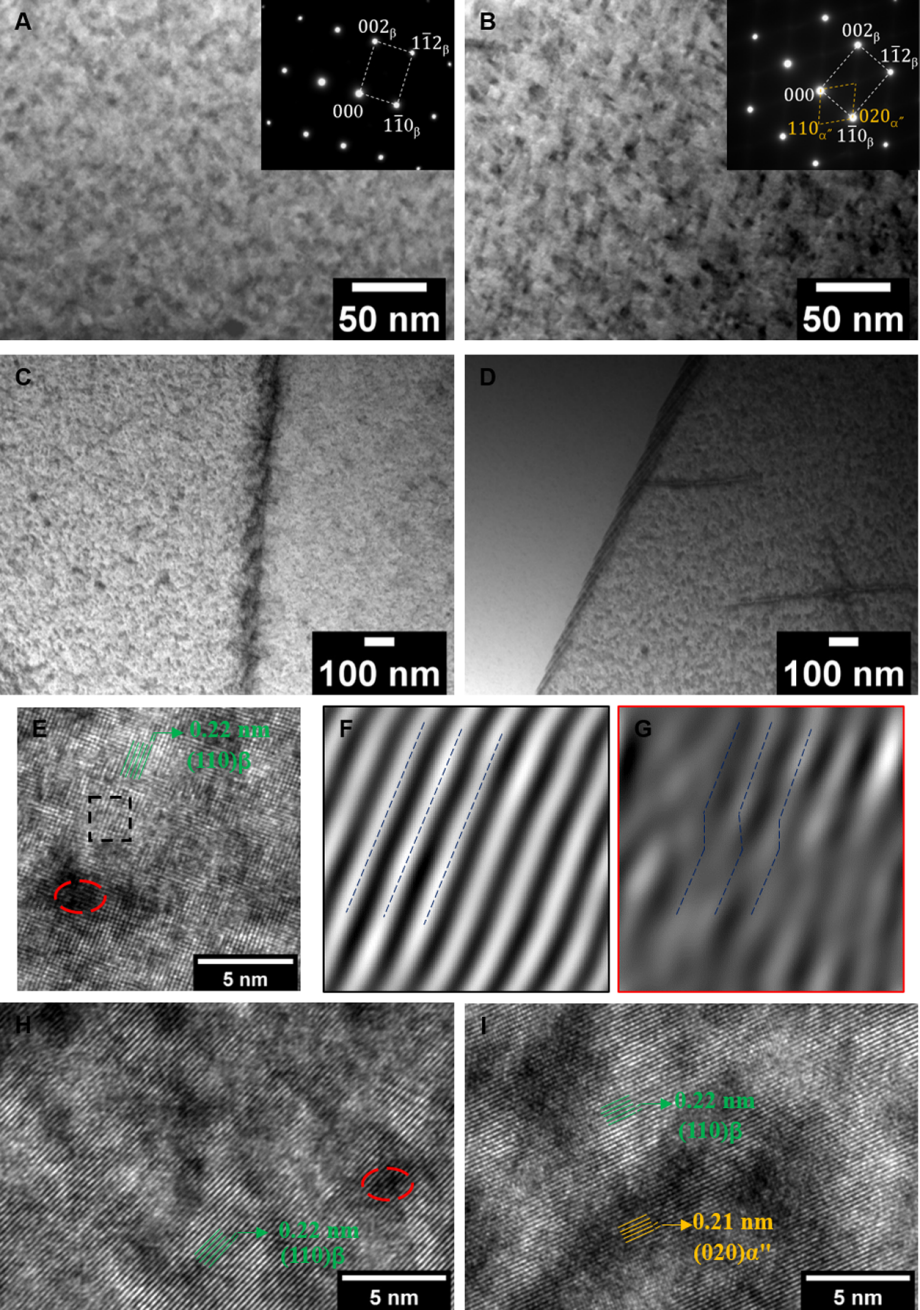
BF images with the corresponding SAD pattern (inset) of (A) TNT5Zr-AF + HIP and (B) TNT5Zr-AF + HIPA, taken from the beta matrix region, viewed from the [110]_β_ zone axis. BF-STEM images of (C) TNT5Zr-AF + HIP and (D) TNT5Zr-AF + HIPA, taken from a region close to the grain boundary. HRTEM images of (E) as-fabricated TNT5Zr alloy and inverse FFT image from the region (F) squared by black dashed lines and (G) circled by red dashed lines. HRTEM images of (H) TNT5Zr-AF + HIP and (I) TNT5Zr-AF + HIPA. Note: the HRTEM images are taken from the [110]_β_ zone axis.

The BF image (Figure 6A) of the TNT5Zr-AF + HIP sample taken from the [110]_β_ zone axis reveals the single beta phase with bcc reflection. Figure 6B showcases the presence of distinguishable ellipsoidal nano-sized precipitates (about 5–10 nm) and a weak reflection from the TNT5Zr-AF + HIPA alloy. It reveals that the orientation relationship between α″ particles and the beta phase can be achieved: (001)_α_″__||(110)_β_. Figure 6C,D presents the BF-STEM images of TNT5Zr-AF + HIP and TNT5Zr-AF + HIPA alloys, which are captured from a region close to the grain boundary. The presence of heterogeneous alpha precipitates can be observed along the grain boundary in the two post-processed TNT5Zr specimens. The HRTEM image taken from the [110] _β_ zone axis (Figure 6E) of the as-fabricated TNT5Zr alloy reveals the presence of the main beta domain (e.g., dashed rectangle) with some inhomogeneously distorted areas (e.g., dashed circle). Inverse fast Fourier transform (IFFT) images show the crystallographic plane difference of as-mentioned domains without or with partial shear (Figure 6F,G). A similar observation can be seen in the TNT5Zr-AF + HIP alloy (Figure 6H). The HRTEM image and IFFT analysis (Figure 6I) indicate that there exist nanoscale local domains with an α″ orthorhombic lattice arrangement in the TNT5Zr-AF + HIPA alloy. The corresponding *d*-spacing calculations suggest that the lattice mismatch between the β and α″ phases is small.

### 3.3 Mechanical Test

**Figure 7:**
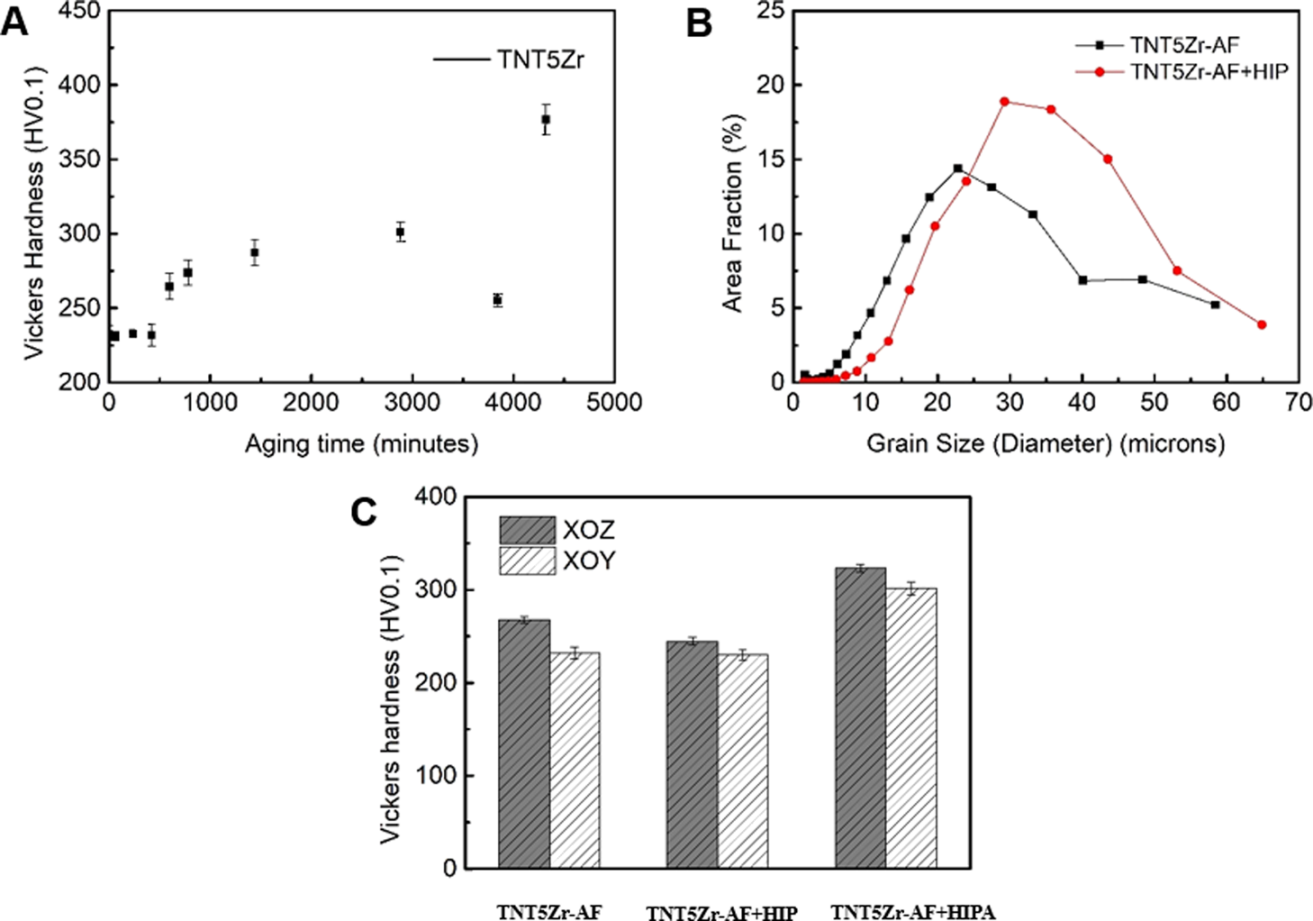
(A) Vickers hardness of the TNT5Zr alloy with different aging time intervals. Note: the specimens are sectioned from the XOY plane. (B) Beta titanium grain size of SLM-manufactured TNT5Zr before and after HIP measured using EBSD. (C) Vickers hardness of different types of manufactured TNT5Zr alloys.

Figure 7A shows the Vickers hardness versus aging time result of the TNT5Zr sample. Overall, the microhardness increase is not severe in the range of a 64 h aging time interval, increasing from 232 ± 6.5 HV0.1 to the maxima (301.3 ± 6.8 HV0.1). It rapidly reached 376.7 ± 10.3 HV0.1 after applying a 72 h aging treatment. The average beta grain size of SLM-manufactured TNT5Zr before and after HIP treatment measured using EBSD quantification is demonstrated in Figure 7B. It can be clearly found that the beta grain size grows after HIP at the temperature above the β-transus. Figure 7C reveals the microhardness homogeneity in both planes sectioned from different types of manufactured TNT5Zr alloys. It can also be found that the slight crystallographic orientation induced a microhardness variation between XOZ and XOY planes (e.g., 267.7 ± 3.4 HV0.1 and 232.0 ± 6.5 HV0.1 in TNT5Zr-AF). The Vickers hardness of the TNT5Zr-AF + HIP sample almost remained at the same level as that of the TNT5Zr-AF alloy. TNT5Zr-AF + HIPA obtained via HIP and 48 h of aging experienced a slight increase (70.1 HV0.1 in the XOY plane) in microhardness compared to the counterpart of TNT5Zr-AF.

**Figure 8:**
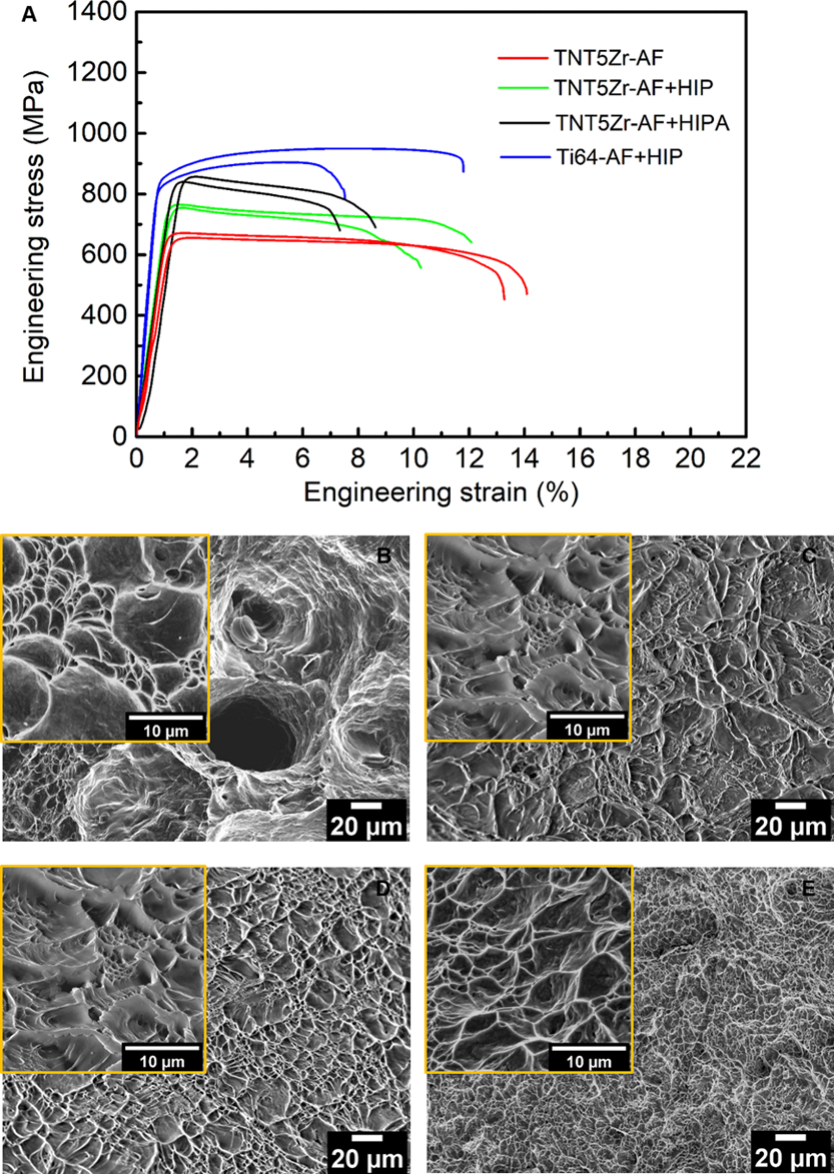
(A) Typical stress–strain curves of different types of manufactured TNT5Zr alloys and post-processed Ti64 specimens. The tensile fracture morphologies for (B) TNT5Zr-AF, (C) TNT5Zr-AF + HIP, (D) TNT5Zr-AF + HIPA, and (E) Ti64-AF + HIP. Note: 1. Here, Ti64 is short for Ti–6Al–4V due to the limited space in the graph. 2. High-magnification SEM images are given in the inset of the corresponding fracture micrographs.

The engineering stress–strain curves of post-processing-treated SLMed Ti alloys are revealed in Figure 8A. Table 2 integrates the values of tensile properties of the involved materials. The most ductile material is TNT5Zr-AF with the lowest UTS of 698 ± 4 MPa and the highest elongation of 13.7 ± 0.6%. TNT5Zr-AF + HIP shows a slightly higher UTS (760 ± 5 MPa) and a lower elongation (11.4 ± 0.7%). The TNT5Zr alloy after HIP and the aging duplex treatment has the highest UTS (853 ± 9 MPa) but sacrifices the material’s plasticity (7.3 ± 1.1%). When comparing the tensile properties of TNT5Zr-AF + HIPA and the reference material (Ti64-AF + HIP), it is found that the TNT5Zr alloy after the duplex treatment has a roughly comparable UTS to that of Ti64-AF + HIP (926 ± 23 MPa). It is noteworthy that TNT5Zr -HIPA still maintains the low Young’s modulus (57 ± 5 GPa), which remains approx. half of the reference material (Ti64-AF + HIP). Figure 8B–E demonstrates the tensile fractures of different types of the manufactured TNT5Zr alloy and the reference material. Figure 8B shows ductile fracture with as-fabricated pores in the TNT5Zr-AF specimen, and large irregular shear-like oval dimple features can be observed in the high-magnification SEM micrograph. Regularly shaped dimples without SLM-induced pores appear in TNT5Zr-AF + HIP (Figure 8C) and TNT5Zr-AF + HIPA (Figure 8D). By comparison, the dimple size is smaller in the Ti64-AF + HIP alloy (Figure 8E) than in the counterparts of TNT5Zr-AF and TNT5Zr-AF + HIP.

**Table 2:** Comparison of the Tensile Properties for Different Types of Manufactured TNT5Zr Alloys and the Ti64-AF + HIP Alloy

material	*E* (GPa)	σ_0.2_ (MPa)	σ_UTS_ (MPa)	δ (%)	σ_UTS_/*E*
TNT5Zr-AF	57 ± 5	650 ± 8	698 ± 4	13.7 ± 0.6	12.1 ± 1.1
TNT5Zr-AF + HIP	63 ± 4	734 ± 8	760 ± 5	11.4 ± 0.7	12.2 ± 0.8
TNT5Zr-AF + HIPA	57 ± 5	831 ± 9	853 ± 9	7.3 ± 1.1	15.2 ± 1.4
Ti64-AF + HIP	107 ± 1	842 ± 9	926 ± 23	9.7 ± 2.1	8.7 ± 0.3
TNTZ^11,27,28^	46–80	447–900	545–950		

**Figure 9:**
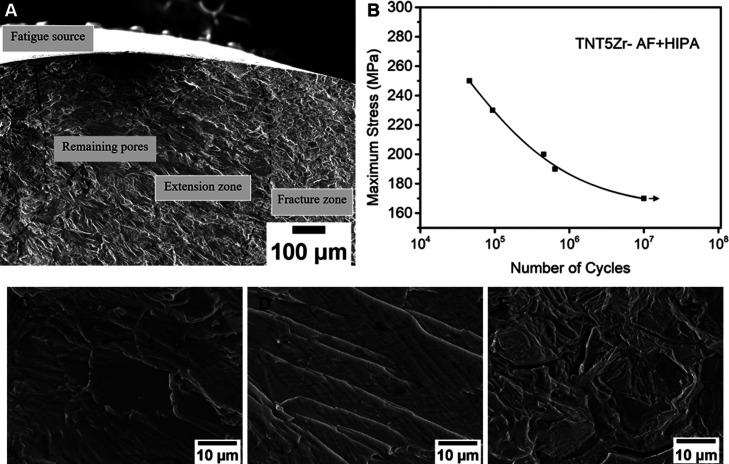
(A) Fatigue fracture with different zones taken from TNT5Zr-AF + HIPA and its (B) *S*–*N* curve. SEM morphology of (C) crack initiation in the fatigue source zone, (D) crack extension zone, and (E) final fast fracture zone. Note: fatigue fracture was obtained from the maximum stress of 200 MPa, *N* = 453,900.

The fatigue test was performed using the TNT5Zr alloy in the condition with the highest UTS, namely, TNT5Zr-AF + HIPA. Fatigue test results of the TNT5Zr-AF + HIPA alloy are demonstrated in Figure 9. The low-magnification SEM image (Figure 9A) shows relatively flat fracture surfaces, and it roughly partitions three fatigue regions, namely, fatigue source, extension, and fracture zone. Multi-source cracks originated from the specimen surface and then expanded radially toward the inside of the specimen, finally forming a fast fracture region. The *S*–*N* curve (Figure 9B) indicates that the TNT5Zr-AF + HIPA alloy possessed a low fatigue life when loading at the level of 230–250 MPa maximum stress. The maximum stress (170 MPa) did not fail at 1 × 10^7^ cycles, which was defined as the fatigue limit of the TNT5Zr-AF + HIPA alloy. Figure 9C reveals its SEM micrograph with a fine extruded ribbon-like formation near the crack initiation site; Figure 9D demonstrates that obviously coarser slip-band cracks propagate along the stable fatigue extension region. Intergranular cracks occur in coarse grain zones, which suffer from cyclic stress and are found at the fast fracture zone (Figure 9E). Fine dimples and micro-cleavage facets are also observed, and no river pattern is formed accordingly.

### 3.4 Biocompatibility Evaluation

**Figure 10:**
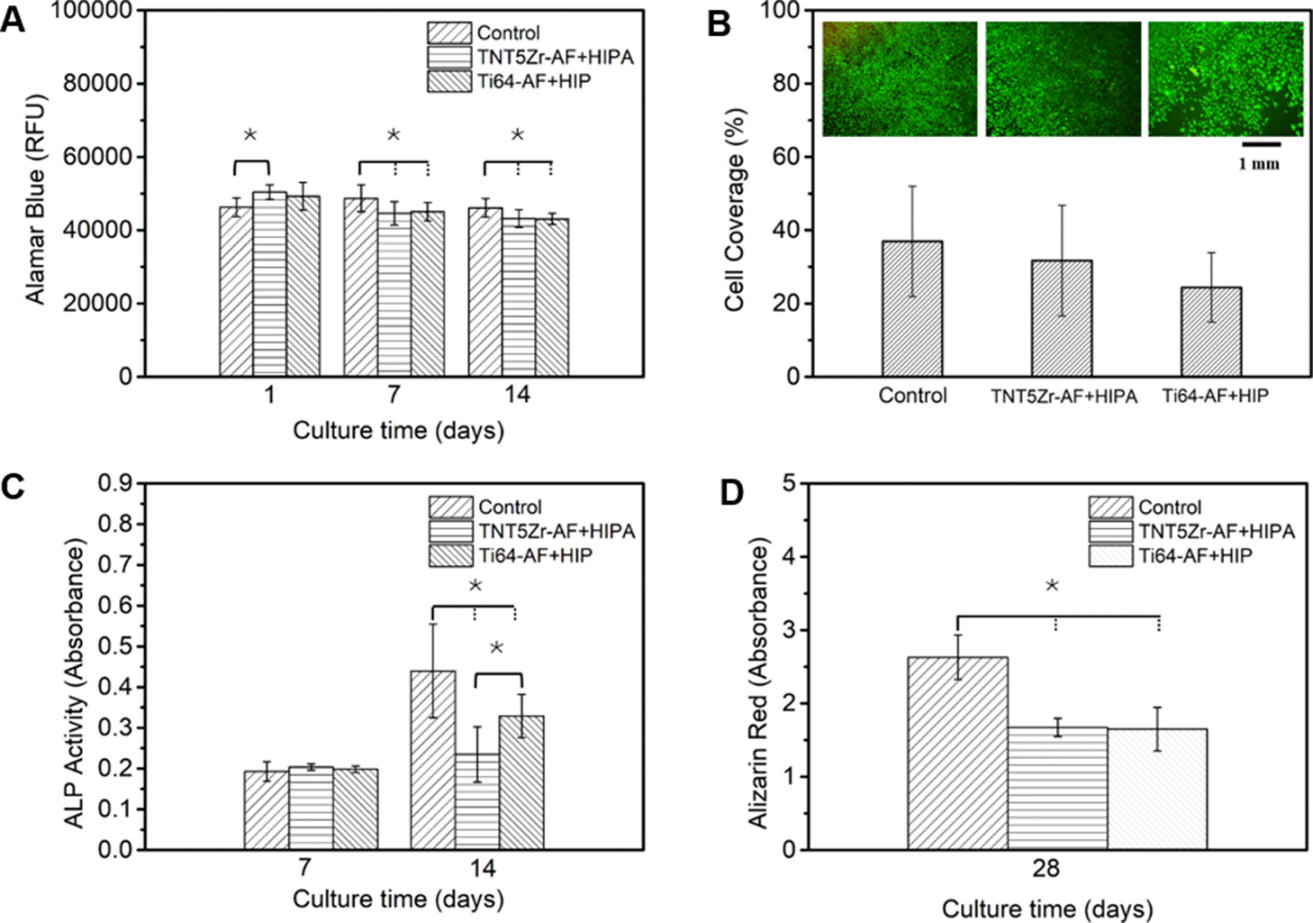
(A) Metabolic activity of MC3T3-E1 preosteoblasts seeded on different substrates. (B) Live cell coverage quantification values and viability staining images after 7 days of culture. (C) Levels of ALP activity measured after different culture time intervals. (D) ARS absorbance values at 28 days post-seeding. Here, * signifies *p* value <0.05 (where more than one pair is illustrated, the comparison between the group is indicated with a solid line and that with any other groups is indicated with dashed lines).

Metabolic activity of MC3T3-E1 preosteoblast cells after 1, 7, and 14 days of incubation within Ti alloy substrates and the plastic control is shown in Figure 10A. A similar metabolic activity was observed after culturing with TNT5Zr-AF + HIPA and Ti64-AF + HIP substrates in these time intervals. In addition, no obvious Alamar blue reduction difference in these groups was observed when the incubation time was increased from 1 day to 14 days. 7 day cell viability fluorescence imaging (Figure 10B) reveals that both metallic surfaces and the plastic control displayed confluent cell growth with no visible damaged membranes. The quantitative values of the cell adhesion area on these substrates show no significant difference after 7 days of culture, and the highest cell coverage was observed in the control group. Figure 10C shows short-term mineralization after 7 and 14 days of culture with different substrates. The fluorescence intensities after 7 days of culture with the two metallic substrates remain at a similar level in comparison with that of the control group. According to 14 day ALP activity results, an increase of the MC3T3-E1 cell differentiation level was observed on both metallic substrates compared to the 7 day counterpart, and the ALP activity values of two Ti alloys were significantly lower than the value in the control group. ARS was used for the examination of long-term (28 days) mineralization deposited on Ti alloy substrates. As shown in Figure 10D, levels of calcium deposits are not statistically different between TNT5Zr-AF + HIPA and Ti64-AF + HIP alloys but are significantly lower than the counterpart collected from the plastic control.

## 4 Discussion

### 4.1 Microstructural Evolution and Defects Distribution of SLM-Manufactured TNTZ Alloys before and after Post-Processing

According to the results of the TNT5Zr-AF alloy, the microstructure of columnar and equiaxed fine grains with a single phase (bcc structure) was retained at room temperature. The EBSD results (Figure 3) have shown that these fine grains possessed a ⟨001⟩ fiber texture. As the component coined in a layer-by-layer consolidation manner, it was determined that grains prefer to grow along the main heat dissipation direction (build direction). No obvious metastable phase peaks such as α″ or ω are observed in the XRD pattern (Figure 4). Meanwhile, no distinguishable acicular α′ phases are observed in its SEM micrograph (Figure 5). The same XRD patterns and SEM micrographs were also observed in the Ti–Nb-based beta titanium alloy with enough β stabilizer elements manufactured using the solution and quenching treatment.^29,30^ The dominant bcc β phase without metastable phases was retained in the grain matrix of the TNT5Zr-AF alloy even though it underwent a high cooling rate in SLM,^31,32^ which demonstrates that the commonly found cooling rate-dependent martensitic transformation is suppressed in our high β-stabilized Ti alloy. The HRTEM image (Figure 6E) taken from the TNT5Zr-AF alloy reveals the lattice spacing of 0.22 nm corresponding to {110} planes of the bcc β phase. The IFFT analysis confirms that no existence of α″ or ω phases was observed, which normally form by moving small distances relative to the unit cell dimensions of the β phase or collapsing two {111} planes of the parent phase into one plane at an intermediate position.^33,34^ As shown in TNT5Zr-AF HRTEM observations (Figure 6E–G), the planar nano-sized areas of the partial plastic shear is considered as nanodisturbances, which has been found in low resistance-to-shear planes in the deformed gum metal.^35^

The HIP treatment broke up the as-fabricated ⟨001⟩ fiber texture and produced the coarser beta grains caused by grain boundary migration when a dwell occurred at the temperature above β-transus. TEM results (Figure 6A,C) indicate the existence of the single beta matrix and alpha precipitates along the grain boundary in the TNT5Zr-AF + HIP alloy. The similar microstructure can be found in homogenized and furnace cooled TNTZ alloys consisting of the β phase and grain boundary α precipitates.^36,37^ The diffusion-controlled coarsening^38^ of precipitates located at the grain boundary in the TNT5Zr-AF + HIP alloy is due to the solute-lean β stabilizers in that zone, thus forming intergranular α precipitates. These particles undergo a further particle size increase after a relative long time furnace cooling.

The TEM investigation of the TNT5Zr-AF + HIPA alloy has shown that ellipsoidal nano-sized α″ precipitates were obtained in the beta matrix, along with intergranular alpha precipitates (Figure 6B,D). The similar microstructure with fine-scale intragranular α″ precipitation was also observed in the Ti–32Nb–(2,4)Sn alloy after low-temperature aging at 400 °C for 2 h.^39^ The nano-sized α″ precipitate formation is accompanied by diffusion-controlled local solute compositional fluctuation at low temperature, which is regarded as the same thermally activated formation mechanism with isothermal ω phases.^40^ According to the fundamental basis of spinodal decomposition,^34^ localized fluctuations in the composition can be triggered in the supersaturated stage and spontaneously grow in the “critical nuclei” in the vicinity via the continuous low-amplitude composition fluctuations, thus gradually evolving into a distinct two-phase mixture. In our case, combining the results that microhardness values did not experience a large increase in the first 48 h of aging treatment (Figure 7A) and ultrafine nano-sized precipitates (approx. 5–10 nm) were obtained in the beta phase matrix (Figure 6B), we presume that the activation energy for the thermally activated diffusional jumps from the parent phase to the critical nucleus during isothermal aging is high in this high β*-*stabilized Ti alloy.

Reconstructed 3D visualization of the keyhole (Figure 2A) from the as-fabricated TNT5Zr alloy is observed via micro-CT. The mechanism of keyhole formation is explained as follows: the rear part of molten pool bears with intensive local evaporation due to the incident beam, and then, the dynamic recoil pressure of the vapor jet and surface tension pressure dent the adjacent wall, leading to keyhole formation.^21,41^ As the TNT5Zr alloy possesses a narrow temperature gap between the highest-melting-point element Ta (3017 °C) and the lowest-boiling-point element Ti (3287 °C), the keyhole formation risk could be high due to the local element evaporation that happens inside the molten pool. It is noteworthy that HIP thoroughly closed these as-fabricated pores. This has been explained by Atkinson and Davies,^23^ who state that the surrounding matter is transported to fill internal pores with the aid of the high-pressure argon atmosphere. However, to fulfill the pore closure at high temperature, the cooling rate-dependent microstructural evolution inevitably occurred in this alloy. In our former work,^20^ micro-CT evaluation showcased an extremely low volume fraction (0.0030%) of unmelted particles (Nb and Ta) in the as-fabricated TNT5Zr alloy. Additionally, no diffraction peak belonging to these unmelted particles can be detected in this alloy (Figure 4). We consider that the microstructural inhomogeneity caused by these negligible particles was not formed due to the high energy input and remelting effect in laser scanning.

### 4.2 Mechanical Properties

The low Vickers hardness value obtained from the as-fabricated TNT5Zr (Figure 7C) is consistent with that of the Ti–24Nb–4Zr–8Sn alloy manufactured via SLM.^42^ Similarly, the tensile test data (Table 2) reveal that the lowest UTS (698 ± 4 MPa) and Young’s modulus (57 ± 5 MPa) were obtained in the as-fabricated TNT5Zr specimen. Slip has been reported in bcc polycrystalline materials on various planes, for example, {110}, {123}, and {112}, containing the ⟨111⟩ direction of close packing,^43^ which enables the as-fabricated TNT5Zr alloy to possess a relatively low critical resolved shear stress. The grain size decided by the Hall–Petch relationship is another factor to explain the flow stress difference of the polycrystalline β Ti alloy under plastic deformation. The available literature^44,45^ show that a comparable UTS (∼680 MPa) is found in the fine-grained Ti–30Nb–5Ta–3Zr alloy manufactured using SLM, and an even lower UTS (449 MPa) is obtained in the coarse-grained Ti–30Nb alloy after cold rolling followed by solution treatment at 750 °C. In addition, it has been found that nanoscale phases play a crucial role in the formation of the unique mechanical properties of the gum metal.^35^ The theory involves that elastic interaction between the nanoscale phasess and provides for the softening of gum metal where they carry plastic flow, which is assumed to be another factor to explain the good plasticity of the as-fabricated TNT5Zr alloy.

Through comparison with the mechanical property results of the TNT5Zr-AF alloy, it retains the same microhardness level but slightly higher UTS after HIP treatment (Table 2). The HIP-treated material obtained a microstructure with coarser beta grains and grain boundary α precipitates. The strengthening induced by grain boundary precipitation and the change of the grain orientation, together with material softening caused by grain growth after the HIP treatment, dominate the resulting flow stress of TNT5Zr-AF + HIP. In comparison with the morphology and size difference of dimples in TNT5Zr-AF and TNT5Zr–HIP alloys, large irregular shear-like and smaller regularly shaped dimples were obtained, respectively. The regularly shaped dimples were observed in the latter alloy because a higher extent of microvoid coalescence occurred in the heterogeneous microstructure of relatively coarse grains with a weaker preferred orientation and grain boundary precipitates. It is noteworthy that no obvious improvement of plasticity of the TNT5Zr-AF + HIP alloy was obtained after pore closure, suggesting that the microstructure is potentially regarded as the more crucial factor than porosity in the determination of its plasticity.

HIP and the aging duplex treatment further strengthen the TNT5Zr alloy by introducing ellipsoidal nano-sized secondary alpha precipitates in the beta matrix (Figure 6B). The nanoscale particles inevitably cause lattice distortions and impede the dislocation movement through a lattice containing precipitate particles. Therefore, the microstructural evolution after the duplex treatment enables the TNT5Zr-HIPA alloy to obtain a comparable UTS (853 ± 9 MPa) to that of Ti64-AF + HIP (926 ± 23 MPa). Moreover, Young’s modulus of the TNT5Zr-HIPA alloy remains as low as that of the TNT5Zr-AF alloy (57 ± 5 GPa). This is inconsistent with the Young’s modulus increases after the aging treatment commonly reported in the literature.^27,46^ The authors suspected that the strengthening induced by fine nano-sized α″ precipitates (about 5–10 nm) in the beta matrix is not severe. In addition, the existence of coarse intercrystallite alpha phases makes grain boundaries probably undergo fast collapse because of crack propagation paths during tension. Overall, the highest ratio of σ_UTS_/*E* (15.2 ± 1.4) of the TNT5Zr-AF + HIPA alloy among the involved materials demonstrates the potential for load-bearing implant application. This strengthened beta Ti alloy possesses an advantage on the “stress shielding” effect reduction compared to the high-elastic-modulus biomedical Ti–6Al–4V.^47^

After studying the fractographic characteristics of different regions (Figure 9) in the TNT5Zr-AF + HIPA alloy, inferior notch-like surface irregularities are regarded as fatigue crack initiation sites. Meanwhile, slip-band cracking has been widely observed in this ductile alloy, caused by cyclic stressing close to the fatigue limit (170 MPa). The ribbon-like extrusion along slip bands normally starts after a small number of loading cycles. The phenomenon of intrusions and extrusions is a result of persistent slip bands beneath the surface. Smaller crack spacing in the crack initiation region demonstrates that the crack growth rate is smaller than in the crack propagation zone. After cracks further aggregate inside the extension region, the crack spacing becomes wider as an indication of local plastic deformation.^48^ When cracks migrate to the final fast fracture region, two crack growth mechanisms occur. Micro-cleavage involves facture along the grain, with coarse intergranular precipitates impeding the gliding and thus providing cracking paths. Due to the stress level of crack propagation along boundaries being probably lower than that of slip-band cracking within grains, the destructive cyclic stress promptly peels off grain boundaries. Moreover, microvoid coalescence that takes place during plastic deformation can be attributed to the microstructure of nano-sized precipitation inside the beta matrix.

The *S*–*N* curve (Figure 9B) shows an intermediate fatigue limit of 170 MPa in the condition of TNT5Zr-AF + HIPA. According to the results from the literature, the measured fatigue limit is lower than the counterpart of as-forged + solution treated Ti–29Nb–13Ta–4.6Zr (320 MPa)^46^ but remains on the same level as that of as-SLMed Ti–30Nb–5Ta–3Zr (140 MPa) obtained using pre-alloyed feedstock.^44^ Here, the improvement of the fatigue limit of the TNT5Zr alloy manufactured using SLM is discussed. First, microstructure tailoring to enhance the strength and ductility of the material can be considered. From the aforementioned tensile testing results, the heterogeneous intergranular second phase (alpha precipitates) induced from the high-temperature HIP treatment deteriorated the ductility of TNT5Zr-HIPA, making cracks propagate preferably along the grain boundary. These precipitates should be suppressed by post-processing treatments undergoing a higher cooling rate. In addition, a proper longer aging time is supposed to increase the content and size of isothermal precipitates inside the beta matrix, enabling dislocation bowing to dominate around particles during plastic deformation. Second, as the nature of the sample surface strongly affects the fatigue initiation and propagation behavior, test pieces with a better surface finish for the fatigue test can significantly enhance the fatigue strength. Unlike the aforementioned fatigue test in the former work^44,46^ using machined test pieces, the authors in this study measured the fatigue limit of the surface-ground samples to better reveal the real surface imperfections fabricated via AM. Surface roughness modification (e.g., chemical polishing)^49,50^ can be introduced to decrease the surface discontinuities of complex-shape AMed components, which helps reduce the stress concentration and further lower the crack initiation risk from the surface. At the same time, recorded data^48^ also revealed that the square cross-section caused a larger decrease in the fatigue limit than circular ones; UTS enhancement caused by the specimen cross-sectional area increasing was observed in as-built Ti–6Al–4V for both cylindrical and rectangular shaped specimen types.^51^ It means that the types of test pieces and the amount of the surface area manufactured via SLM should be considered well.

### 4.3 *In Vitro* Performance

The biocompatibility investigation (Figure 10) according to quantitative results has shown almost no significant difference in cell–substrate (metallic ones) interactions, in terms of viability, differentiation, and mineralization. Similarly, it has shown the same level of MC3T3-E1 preosteoblast attachment, spreading, and proliferation between Ti–23Nb–7Ta–2Zr–0.5N alloy and Ti–6Al–4V control samples.^16^ The passive oxide films formed from TNT5Zr and Ti–6Al–4V alloys presumably exhibit subtle chemistry differences in these short-term cultures; thus, negligible toxicity was observed due to the protection of various oxides (Figure 10A). The available literature^52^ found that the quantities of alloying elements (Ti, Al, and V) released from the Ti–6Al–4V alloy and alloying elements (Ti, Zr, Nb, and Ta) from the Ti–15Zr–4Nb–4Ta alloy were negligible after immersion in the cell culture medium for 1 week. However, the metallic ion concentration as a function of the *in vivo* implantation period (0–48 weeks) result revealed a metallic ion level increase in Ti–6Al–4V but a much lower released ion level in the latter alloy. As cytotoxicity induced from released metallic ions is crucial for long-term implantation of any biomaterial,^13^ the more noble corrosion-resistant TNTZ alloy may become an advantageous Ti alloy for use, which is also reported in Atapour et al.^53^ The 14 day ALP content and 30 day calcium deposit quantification suggests a significantly lower level of cell differentiation than the counterpart of the control group. These results without normalization ignored the surface area of substrates. Because preosteoblasts cultured in the plastic control substrate possessed a 2.5 times larger surface area than the counterpart of the two Ti alloy substrates, a comparable level of the mineralization results can be obtained after proper normalization. During *in vitro* MC3T3-E1 tests, the authors consider that the SLM-manufactured TNT5Zr can be used as a biomaterial similar to the well-accepted bio-friendly Ti–6Al–V.

## 5 Conclusions

This study investigated the microstructural evolution, defects distribution, and mechanical properties of the SLM-manufactured TNT5Zr β Ti alloy before and after the post-processing treatment. In addition, we evaluated the short-term *in vitro* MC3T3-E1 preosteoblast response of the post-processed β Ti alloy and Ti–6Al–4V alloy. The following main conclusions are drawn:

1. HIP treatment closes the as-fabricated keyhole pores in the TNT5Zr alloy due to densification caused by the pressured and high-temperature argon atmosphere. Meanwhile, the grain growth and slighter extent of the preferred crystallographic orientation are observed in the specimen after HIP. The BF image and SAD pattern together with the BF-STEM observation show the existence of the single beta matrix and alpha precipitates along the grain boundary in the TNT5Zr-AF + HIP alloy. The same microstructure including ellipsoidal nano-sized α″ precipitates (about 5–10 nm) in the β matrix is obtained in the TNT5Zr-AF + HIPA alloy.
2. By comparison with the mechanical properties of TNT5Zr-AF alloy, it is shown that a slightly higher UTS (760 ± 5 MPa) remains after the HIP treatment. HIP and the aging duplex treatment further strengthen the ductile TNT5Zr alloy via precipitation strengthening, enabling it to obtain a comparable UTS (853 ± 9 MPa) to that of Ti64-AF + HIP (926 ± 23 MPa).
3. Inferior notch-like surface irregularities are regarded as fatigue crack initiation sites in the TNT5Zr-AF + HIPA alloy. Slip-band cracking has been widely observed in this alloy, caused by cyclic stressing close to the fatigue limit (170 MPa). Both crack growth mechanisms, namely, micro-cleavage and microvoid coalescence, take place when cracks migrate to the final fast fracture region.
4. *In vitro* biocompatibility investigation of both Ti alloys has shown similar metabolic activity and long-term mineralization. The high strength-to-modulus ratio (15.2 ± 1.4) together with the excellent biological *in vitro* behavior demonstrates that the TNT5Zr-AF + HIPA alloy can be a good candidate for a load-bearing implant.
